# Identifying key predictors for uterine manipulator use in robotic simple hysterectomy: a retrospective cohort analysis

**DOI:** 10.3389/fmed.2024.1462632

**Published:** 2024-09-11

**Authors:** Shogo Kawamura, Kuniaki Ota, Yoshiaki Ota, Toshifumi Takahashi, Hitomi Fujiwara, Keitaro Tasaka, Hana Okamoto, Yumiko Morimoto, Wataru Saito, Mika Sugihara, Takehiko Matsuyama, Eiji Koike, Mitsuru Shiota, Koichiro Shimoya

**Affiliations:** ^1^Department of Obstetrics and Gynecology, Kawasaki Medical School, Okayama, Japan; ^2^Fukushima Medical Center for Children and Women, Fukushima Medical University, Fukushima, Japan; ^3^Department of Obstetrics and Gynecology, Koujin Hospital, Kagawa, Japan; ^4^Department of Obstetrics and Gynecology, Koike Hospital, Hiroshima, Japan

**Keywords:** robotic simple hysterectomy, robot-assisted simple hysterectomy, uterine manipulator, da Vinci Xi surgical system, pouch of Douglas obliteration, operative assistant

## Abstract

**Background:**

Robotic simple hysterectomy (RSH) is the most common robotic gynecologic surgery in the United States. Uterine manipulators are commonly used to handle the uterus during laparoscopic surgery, but few studies have examined their necessity in RSH. This study retrospectively compares RSH cases with and without the use of manipulators, and identifies predictors for their intraoperative use.

**Materials and methods:**

This retrospective cohort study included patients undergoing RSH for benign pathologies at Kawasaki Medical School from October 2020 to December 2022. Patients with malignancies were excluded. The robotic surgeries were performed by three skilled surgeons using the four-arm da Vinci Xi surgical system. Data on perioperative and operative parameters were collected, including age, body mass index (BMI), history of abdominal surgery, disease type, presence of ovarian cysts, and operative time. Statistical analyses were performed using EZR software, with multivariate logistic regression to identify predictive factors for uterine manipulator use.

**Results:**

The study included 113 patients who underwent RSH without a uterine manipulator and 58 with one. Patients without a manipulator were older, while those with a manipulator had higher BMIs and a higher prevalence of ovarian chocolate cysts and Douglas obliteration. Operating time was shorter without a manipulator. Independent predictors for manipulator use were higher BMI, presence of ovarian endometrioid cysts, and Douglas obliteration.

**Conclusion:**

RSH without a uterine manipulator is feasible and can reduce the need for surgical assistants. Predictors for manipulator use include higher BMI, ovarian cysts, and Douglas obliteration. The use of a fourth robotic arm can enhance surgical independence and resource efficiency. Further research is needed to assess the long-term cost-effectiveness and outcomes of this approach.

## 1 Introduction

Hysterectomy is the most frequent surgical procedure performed on women with uterine benign diseases accounting for approximately 90% of hysterectomies (1). Before surgical robots, laparoscopy was the only minimally invasive option, limited by its steep learning curve and need for advanced training. Since the United States Food and Drug Administration approved the da Vinci robot (Intuitive Surgical) in 2005, advancements in robotic technology have greatly increased its use in gynecologic surgeries. At present, robotic simple hysterectomy (RSH) is now the most common robotic gynecologic surgeries in the United States (2, 3).

The greatest advantage of robotic surgery compared with laparotomy or laparoscopy is the saving in human resources. The equipment for RSH is slightly more expensive per procedure than for total laparoscopic hysterectomy (TLH), but performing 45 or more RSH procedures becomes more cost-effective than TLH (4, 5). The use of uterine manipulators is well established and it is clear that uterine manipulators offer the easiest way to handle the uterus during surgery (6). TLH without a uterine manipulator has been reported to reduce operative time and the need for a pelvic assistant (7). However, few studies have examined whether manipulators are necessary for RSH. In this study, we aimed to retrospectively compare cases of RSH with and without the manipulator and identify predictors for the intraoperative use of manipulators.

## 2 Materials and methods

### 2.1 Study design and data collection

This study was reviewed and approved by the Human Research Ethics Committee of Kawasaki Medical School (trial registration no.: 5043-03). After institutional review board approval, this study was designed as a retrospective cohort study. Patients who underwent RSH at the Women’s Medical Center, Kawasaki Medical School from October 2020 and December 2022 were included. Inclusion criteria encompassed RSH for benign pathologies, including fibroids, adenomyosis, cervical diseases (such as high-grade cervical intraepithelial neoplasia), and endometrial diseases (such as endometrial hyperplasia without atypia). All patients provided consent before the procedure. Patients with indications of malignancy were excluded from this study.

The robotic surgeries were performed by three skilled surgeons (surgeons A, B, and C). Surgeon A was awarded a class B International license by The Japanese Society for Robotic Surgery and is a proctor in The Japan Society for Endoscopic Surgery and Intuitive Surgical; Surgeon B holds a class B domestic license from The Japanese Society for Robotic Surgery and is a proctor in Intuitive Surgical; and Surgeon C is a proctor in Intuitive Surgical. Each surgery was assisted by a gynecological resident who had at least 1 year of experience in robotic surgeries, having participated in more than 30 cases.

We collected the data regarding perioperative parameters: age, body mass index (BMI), history of abdominal surgery, types of disease, presence of ovarian chocolate cysts, presence of pouch of Douglas obliteration, and operative parameters as follows: estimated blood loss, operating time defined as the time from skin incision to skin closure for RSH, concomitant procedures, and uterine weight excised.

### 2.2 Surgical procedures

Under general anesthesia, the patient was placed in a lithotomy position with Trendelenburg tilt. All robotic procedures utilized the four-arm da Vinci Xi surgical system (Intuitive Surgical Inc., Sunnyvale, CA, USA). Initially, an 8-mm endoscope port trocar was inserted 3 cm above the umbilicus using the direct closed method to establish pneumoperitoneum. Three 8-mm robotic ports were then inserted under direct vision on the same horizontal line, spaced 8 cm apart at the level of the endoscope port. The second arm managed the endoscope, while the first arm operated fenestrated bipolar forceps and the third arm managed Maryland bipolar forceps using the double bipolar technique (8). The fourth arm utilized Cadiere forceps to manipulate the uterus, eliminating the need for a uterine manipulator. Suturing was performed using the Suture Cut needle driver on the Maryland bipolar forceps on the third arm, which enabled both suturing and cutting of threads. For suction and intra-abdominal needle transport, the Probe Plus II (Ethicon, Tokyo, Japan) was introduced through the third arm. However, when it was necessary to pull the uterus or bowel toward the head to ensure a clear surgical field, a uterine manipulator was used.

The hysterectomy procedure followed our standard operating procedure for conventional RSH (9). Briefly, the round ligament was transected initially, followed by dissection of the broad ligament anteriorly and posteriorly using the double bipolar method with the Maryland bipolar and fenestrated bipolar forceps. Dissection of the bladder from the proximal vagina was followed by incision and expansion of the peritoneum of vesico-uterine pouch to identify and ligate the ureter and uterine artery, including the ureter-uterine artery crossover point, with 2-0 Vicryl (Ethicon, Tokyo, Japan) sutures. The uterine artery and ascending branches of the uterine vessels were ligated at two points with C 2-0 Vicryl (Ethicon, Tokyo, Japan) and coagulated using the fenestrated bipolar forceps before transecting with the Maryland bipolar forceps. The cardinal ligaments were transected, and colpotomy was performed with the Maryland bipolar forceps, followed by vaginal extraction of the uterus. A large uterus was divided into removable segments and extracted vaginally. Closure of the vaginal cuff was achieved using interrupted 0-Vicryl (Ethicon, Tokyo, Japan) sutures. A video clip summarizing the RSH technique is available (Supplementary Video 1).

### 2.3 Statistical analysis

Statistical analyses were performed using EZR (Saitama Medical Center, Jichi Medical University, Saitama, Japan), a graphical user interface for R (The R Foundation for Statistical Computing, Vienna, Austria) (10). Data was represented as median and interquartile ranges (IQR) for non-parametric variables, and categorical variables are described as frequency and percentage and compared between the groups using Mann–Whitney’s U test (for numeric non-parametric variables) or Fisher’s exact test (for categorical variables). Multiple logistic regression analyses were performed to investigate potential influencing factors to evaluate RSH with a uterine manipulator, employing a forward stepwise methodology to identify independent predictive factors. Specifically, odds ratios (ORs) and 95% confidence intervals (CIs) were calculated in the multivariate analyses, controlling for potential confounders such as age, BMI, history of abdominal surgery, types of diseases, location of fibroids, console surgeons, presence of ovarian endometrioid cysts, presence of pouch of Douglas obliteration, and extracted uterine weight. These factors relate to securing the operative field before or at the start of surgery. Factors such as operative time and operative blood loss, which occur after surgery begins, were excluded as adjustment factors because they were not related to whether manipulators were used. However, for console surgeons, there was a significant difference in the number of RSH surgeries among surgeons. Therefore, console surgeon was included as an adjustment factor. Statistical significance was set at *P* < 0.05.

## 3 Results

The characteristics of patients who underwent RSH without a manipulator (113 patients) and RSH with a manipulator (58 patients) are summarized in Table 1. Patients in RSH without a manipulator were significantly older than those in RSH with a manipulator (median 48.0, IQR 45.0–51.0 versus median 46.5, IQR 42.3–50.0, *P* = 0.04), and BMI was significantly higher in RSH with a manipulator than in RSH without a manipulator (median 25.7, IQR 22.6–29.0 versus median 23.8, IQR 20.8–27.1, *P* = 0.02). There were no statistically significant differences in the types of benign disease excluding the presence of cervical or broad ligament fibroid between the groups. The percentage of cervical or broad ligament fibroids was significantly higher in the RSH with a manipulator group than that in RSH without a manipulator group (13.8 versus 4.4%, *P* = 0.03). There were no statistically significant differences in the history of abdominal surgery between the groups. The percentage of presence of ovarian chocolate cysts was significantly higher in the RSH with a manipulator group than in the RSH with a uterine manipulator group (22.4 versus 3.5%, *P* < 0.01). The percentage of pouch of Douglas obliteration was significantly higher in the RSH with a manipulator group than in the RSH with a uterine manipulator group (25.9 versus 1.8%, *P* < 0.01).

**TABLE 1:** Patient characteristics in both RSH without and with a uterine manipulator.

	RSH without a uterine manipulator (*n* = 113)	RSH with a uterine manipulator (*n* = 58)	*P*-value
Age, years	48.0 (45.0–51.0)	46.5 (42.3–50.0)	0.04
BMI, kg/m^2^	23.8 (20.8–27.1)	25.7 (22.6–29.0)	0.02
**Types of diseases**			
Fibroids, number (%)	73 (64.6)	39 (67.2)	0.73
Adenomyosis, number (%)	32 (28.3)	16 (27.6)	0.92
Cervical disease, number (%)	3 (2.7)	2 (3.4)	1.00
Endometrial disease, number (%)	5 (4.4)	1 (1.7)	0.67
Presence of ovarian endometrioid cyst, number (%)	4 (3.5)	13 (22.4)	< 0.01
Presence of pouch of Douglas obliteration, number (%)	2 (1.8)	15 (25.9)	< 0.01
History of abdominal surgery, number (%)	7 (6.2)	5 (8.6)	0.56

Data are presented as median (interquartile range) or number (percentage). RSH, robotic simple hysterectomy; BMI, body mass index.

The surgical outcomes of RSH without a uterine manipulator and RSH with a uterine manipulator are summarized in Table 2. Operating time was significantly shorter in RSH without a uterine manipulator than in RSH with a uterine manipulator (125.0, IQR 112.0–138.0 versus 148.5, IQR 133.0–172.5). Both estimated blood loss and uterine weight were not significantly different between the groups. For console Surgeon A, the percentage of RSH with a uterine manipulator was significantly higher than that of RSH without a uterine manipulator (65.6 versus 38.9%, *P* < 0.01). For console Surgeon B, the percentage of RSH with a uterine manipulator was significantly lower than that of RSH without a uterine manipulator (3.4 versus 48.7%, *P* = 0.02). For console Surgeon C, there was no significant difference in the percentage of RSH with and without a uterine manipulator.

**TABLE 2:** Surgical outcomes in both RSH without and with a uterine manipulator.

	RSH without a uterine manipulator (*n* = 113)	RSH with a uterine manipulator (*n* = 58)	*P*-value
Operating time, min	125.0 (112.0–138.0)	148.5 (133.0–172.5)	< 0.01
Estimated blood loss, ml	25.0 (10.0–53.0)	37.5 (10.0–69.0)	0.18
Uterine weight, g	182.0 (138.0–260.0)	211.0 (135.5–364.0)	0.28
**Console surgeons, number (%)**			
A	44 (38.9)	38 (65.5)	< 0.01
B	55 (48.7)	17 (3.4)	0.02
C	14 (12.4)	3 (5.2)	0.14

Data are presented as median (interquartile range) or number (percentage). RSH, robotic simple hysterectomy.

Predictive factors for uterine manipulator use during RSH are summarized in Table 3. Multivariate logistic analysis showed that BMI (adjusted OR: 1.15, 95% CI 1.04–1.26, *P* < 0.01), the presence of ovarian endometrioid cysts (OR: 5.25, 95% CI 1.19–23.10, *P* = 0.03), and the presence of pouch of Douglas obliteration (OR: 32.6, 95% CI 3.73–285.00, *P* < 0.041) were independent predictive factors for uterine manipulator use during RSH. Adjusting explanatory variables, history of abdominal surgery, uterine weight, and cervical or broad ligament fibroids were not significant predictors for uterine manipulator use during RSH.

**TABLE 3:** Predictors of uterine manipulator use during RSH.

	Adjusted odds ratio* (95% confidence interval)	*P*-value
BMI	1.15 (1.04–1.26)	< 0.01
History of abdominal surgery	2.35 (0.56–9.76)	0.24
Uterine weight	1.00 (0.99–1.00)	0.14
Cervical fibroid	2.68 (0.62–11.60)	0.19
Presence of ovarian endometrioid cyst	5.25 (1.19–23.10)	0.03
Presence of pouch of Douglas obliteration	32.6 (3.73–285.0)	< 0.01

*Adjusted for age, body mass index, the types of disease, and the console surgeons. RSH, robotic simple hysterectomy.

## 4 Discussion

In this study, we showed for the first time that independent predictors of uterine manipulator use during RSH were BMI, presence of endometrial cysts in the ovary, and Douglas obliteration.

The use of manipulators for RSH is the technique derived from imitation methods of the TLH. In TLH procedures, several studies have highlighted the importance of uterine manipulators in reducing complications during hysterectomy (6, 11). In fact, a uterine manipulator is routinely used as the gold standard in TLH to allow better exposure of the anatomical spaces, consequently lower the overall complication rate, and to prevent bowel and ureteral injuries (12, 13). On the other hand, recently, the use of uterine manipulators has been increasingly discouraged in laparoscopic and robotic surgery. The use of the uterine manipulator in TLH might be avoided in malignant tumor because it is suggested to increase concerns regarding the dissemination of malignant cells to the vaginal cuff and the peritoneal cavity through the fallopian tubes when the uterine manipulator is used for endometrial carcinoma (14). Therefore, there have been many reports of TLH without manipulators in recent years (15), although TLH without the use of a manipulator is a more demanding surgical procedure (16–19). This is because the learning curve for performing TLH without a uterine manipulator is likely to be longer (20, 21). Because robotic surgery has advantages such as a short learning curve, even in RSH, uterine manipulators tend not to be used for endometrial cancer (22).

Recent studies on uterine manipulators in gynecological surgery provide mixed insights. Ongoing research may provide clearer guidelines for their optimal use in various gynecological surgeries. To date, there is no conclusion to the debate on whether a uterine manipulator should or should not be used for TLH, although recent studies on uterine manipulators in gynecological surgery provide mixed insights. In particular, some reports of surgical approaches for a TLH without a uterine manipulator (23) raised concerns over the use of a uterine manipulator for uterine cancers during TLH (24, 25). The meta-analysis by Scutiero et al. (26) suggests that while manipulators don’t significantly impact overall or disease-free survival in endometrial cancer cases, they may increase the risk of positive peritoneal cytology. The prospective randomized trial found that the use of a uterine manipulator during minimally invasive staging for early-stage endometrial cancer does not significantly impact lymph vascular space invasion or patient outcomes (27). Therefore, uterine manipulators are expected to be in increasing demand. On the other hand, Cianci et al.’s (28, 29) reviews highlight the importance of tailoring surgical approaches to individual cases, especially for fibroid treatment and large uterine. Their study on laparoscopic hysterectomy conversions indicates that uterine size significantly affects procedural success, with manipulators potentially less effective for very large uterine (28, 29). Overall, while uterine manipulators offer benefits in visualization and ease of minimally invasive procedures, their use should be carefully considered based on factors like uterine size, cancer risk, and patient characteristics. Hence, it is the new era to adapt to hysterectomy even if a manipulator was not available. Zygouris et al. (15) concluded the feasible and safe technique using grasping forceps for uterine manipulation instead of a uterine manipulator from a large clinical study, if performed by well-trained, experienced laparoscopic surgeons. Moreover, Abdel Khalek et al. (11) advise that surgeons performing TLH should decide on a case-by-case basis whether a uterine manipulator is necessary and, if so, which type best suits the procedure.

The study by Gallotta et al. (30) demonstrates that robotic surgery (RS) is a feasible and safe option for elderly (65–74 years) and very elderly (≥ 75 years) women undergoing gynecologic oncologic procedures. While their study doesn’t specifically address uterine manipulators, the precision of robotic systems may reduce the need for extensive manipulation, potentially benefiting older patients with more fragile tissues (30). Although there were no elderly patients in this study, it may provide the basis for further development into robotic sacrocolpopexy does not use manipulators if this study has advanced and establish as a manipulator-free RSH.

Robotic surgery is considered as advantageous for reducing human resources, but an assistant is still needed. When a uterine manipulator is used, at least two assistants are required. Additionally, the assistant must work in a confined space with the patient’s legs spread, causing significant stress during surgery. Determining whether a uterine manipulator can be used before or immediately after surgery helps reduce the number of assistants needed for the procedure. Perrone et al. (31) described that RSH with the assistance of the fourth robotic arm instead of a uterine manipulator may reduce the need for uterine manipulation because the operating time was not different from TLH without a uterine manipulator done by the experts. Our technique of RSH without a uterine manipulator is consistent with the very same concept. Recently, Barger et al. (32) described that adding a fourth robotic arm to the standard three port setup can markedly improve robotic hysterectomy without a uterine manipulator. In this study, RSH utilizing four arms also allowed us to complete the hysterectomy without a uterine manipulator; however, even with four arms, a manipulator was necessary in cases with increased BMI, the presence of ovarian endometrioid cysts, and the presence of pouch of Douglas obliteration. In those cases, the console surgeon’s independence may be somewhat diminished, as an additional surgical assistant is required. On the other hand, reducing human resources for surgery would deprive fellows or residents of the opportunity to enter robotic surgery. In fact, Hall et al. (33) reported that few fellows were deemed competent enough to independently operate the robot with only 15% being able to perform an entire hysterectomy. Therefore, recent curriculum for OB/Gyn residents and fellows reduces barriers by providing protected time away from clinical duties to provide a reproducible platform for the early acquisition of advanced robotic skills outside of the operating room to standardize mastery-based training for the next generation of robotic surgeons (34).

The difficulty of performing a hysterectomy increases when fibroids are located in the cervix or the broad ligament. In laparoscopic surgery, where manual traction is not possible, the location of the fibroids particularly affects the surgical difficulty. Our study found no association between the use of uterine manipulators and cervical or broad ligament fibroids when adjusted for explanatory variables. Of the 13 cases with cervical or broad ligament fibroids, eight were performed by Surgeon A, five by Surgeon B, and none by Surgeon C (data not shown). These results may be influenced by differences in surgeon experience and should be interpreted with caution.

Although this is a robot-specific study, it may be necessary to consider whether robotic surgery is more significant than laparoscopic surgery. In a previous review, RSH had a longer operating time than TLH (35). However, in the recent RCT-only meta-analysis, there was no difference in operating time between RSH and TLH, and those are now rated as equivalent operative techniques (36). Both the decade of surgeons’ experience and robotic development realized the democratization and widespread of robotic surgery and might reduce the operating time. However, there is no doubt that robotic surgery will surpass laparoscopic surgery in the future. Although the current challenge lies in the training of surgeons and the development of the operating room of the future, In the era of digital surgery, robotic platforms serve as computer interfaces capable of integrating various real-time data analysis modalities, and the next decade enables advanced systems to provide augmented surgical vision through augmented reality, improved surgical decisions using artificial intelligence, and enhanced surgical maneuvers through the advancement of robotic instruments (37). In addition, recent meta-analysis demonstrates that 3D vision systems offer significant advantages over 2D systems in laparoscopic surgery, particularly in terms of improved depth perception, precision, and task completion time (38). All robotic systems always include 3D vision systems, and could be especially beneficial for the training of surgeons to enhance spatial awareness and precision afforded by 3D vision. Pavone et al. (39) have systematically studied the advantages and advancements of robotic platforms in gynecological surgery, and the potential benefits of robotic approaches are sufficient strength for endometriosis surgery (40) and superior to laparoscopic approaches for severe cases such as deep endometriosis through a meta-analysis (41). Furthermore, those foundations are more established when they combine structured training in skill development, novel techniques such as ultrasound-guided robotic surgical procedures, and the integration of imaging technologies (42, 43). Therefore, the potential benefits in the next decade would also depend on the widespread adoption of robotic surgery systems in gynecological surgery settings emphasizing technological advancements, comparative effectiveness, training methodologies, and the integration of imaging techniques.

Our study had several limitations. First, the data may have incomplete information that was not fulfilled in the patient record because of the retrospective nature of the study, which limits the generalizability of our findings. In this retrospective study, there was the possible allocation biases arising from the retrospective comparison between RSH without a uterine manipulator and RSH with a uterine manipulator because of the non-randomized nature of the study design. Therefore, we further need to analyze with a propensity-matched analysis to decrease biases arising from different covariates if both cases are increased, or further prospective trials are needed to confirm our results. Second, three surgeons from our robotic surgical team performed the surgeries. Although most surgeons in the team were trained at the same institution, biases resulting from individual surgeon differences cannot be excluded. Moreover, the most proficient surgeon (Surgeon A) required a uterine manipulator for RSH, which may have resulted in an unbalanced distribution of surgical difficulty and may have impacted the statistical analysis. Third, In the current landscape, other robotic systems have been developed, and the Hugo™ RAS (Medtronic, Minneapolis, MN, USA) or hinotori™ system (Medicaroid Corporation, Kobe, Japan) for hysterectomy has demonstrated effectiveness (44–46). It has raised the possibility of different results to operate with other robotic systems, although Matsuura et al. (47) described that surgeons who are already proficient in performing robotic surgery with da Vinci X can safely perform surgeries with the new models when three robotic systems were compared. The study’s main strength is its description of a four-arm approach to hysterectomy using the da Vinci Xi without a uterine manipulator, which enabled minimal dependence on an assistant. Although previous reports have shown the superiority of a four-arm hysterectomy approach for malignant gynecological diseases (48), this is the first study to demonstrate the superiority of this hysterectomy approach for benign gynecological diseases. Furthermore, this report seems worthwhile because pelvic occupying diseases, such as enlarged uterine fibroids, are more difficult to treat robotically than with radical total hysterectomy.

In conclusion, the routine use of a fourth robotic arm during RSH provides the operating surgeon with greater independence during critical phases of the procedure without the requirement of a uterine manipulator and assistant. This advantage translates into non-dependence on an assistant and the conservation of human resources as well as medical resources such as a uterine manipulator. Although the initial investment in robotic surgical systems is high, we need further longitudinal research on whether shorter hospital stays, reduced postoperative complications, and quicker recovery times, can significantly lower overall healthcare costs.

## Data Availability

The data analyzed in this study is subject to the following licenses/restrictions: Raw data were generated at Kawasaki Medical School. Derived data supporting the findings of this study are available from the corresponding author YO on request. Requests to access these datasets should be directed to yoshimon@med.kawasaki-m.ac.jp.
